# Cydnidae Pigmentation on Palms Resembling Black-Ink Stains

**DOI:** 10.7759/cureus.62578

**Published:** 2024-06-18

**Authors:** Devayani Pol, Ajay Kumar, Shambhavi Singh, Nishtha Malik

**Affiliations:** 1 Department of Dermatology, Venereology and Leprosy, Dr. D. Y. Patil Medical College, Hospital and Research Centre, Dr. D. Y. Patil Vidyapeeth (Deemed to be University), Pune, IND

**Keywords:** dermoscopy, monsoon, burrowing bug, black-ink stains, cydnidae pigmentation

## Abstract

We report a case of a 26-year-old male, a student by profession, who noticed black-ink-like stains over palms not removed with soap and water on relocating to a new flat during monsoons. The patient had no history of trauma, contact with chemicals, or drug intake. Sudden onset during monsoons, absence of symptoms, characteristic clinical and dermoscopic findings, and spontaneous resolution within 10 days led to a diagnosis of cydnidae pigmentation.

## Introduction

Cydnidae bugs (also termed burrowing bugs) are arthropods of the order Hemiptera recognized by their morphological adaptations for digging. In self-defense, they secrete an unpleasant material from unique glands. When these insects are accidentally crushed or compressed, reddish-brown pigmented macules of varied sizes and shapes emerge quickly at the contact site with this fluid. These macules progressively disappear over the course of 10-15 days [1].

## Case presentation

A 26-year-old male student presented with a history of the sudden appearance of black-ink-like stains over the palmar aspect of both hands two days back after moving to a new flat during the rainy season. There was no history of pain, trauma, redness, or swelling at the site of lesions or drug intake before onset.

**Figure 1:**
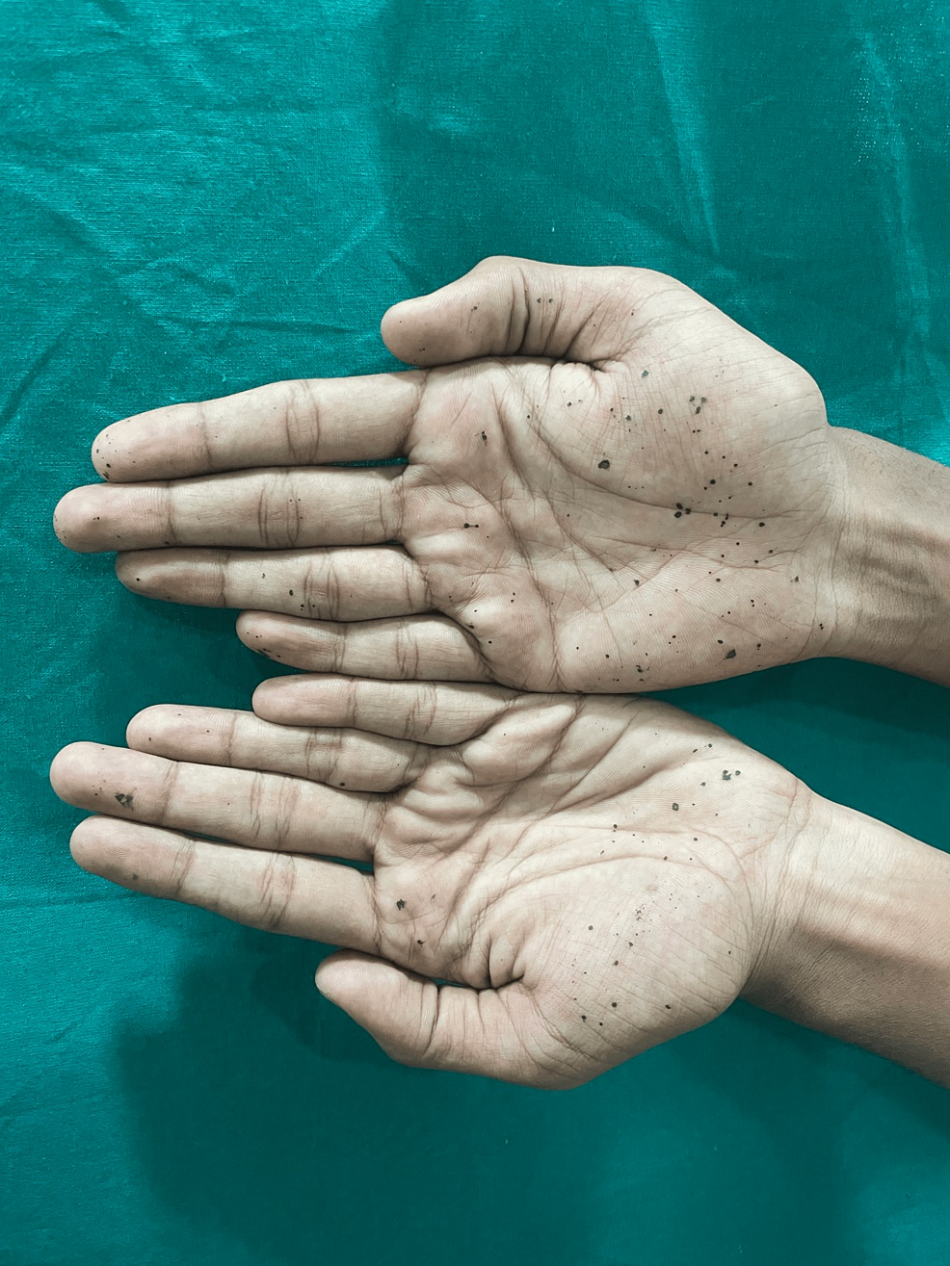
Clinical image showing multiple discrete black macules resembling ink-like stains present on both palms

On clinical examination, well-defined, nonblanchable, nonscaly, nontender discrete black macules of varying sizes and shapes were present on both palms (Figure 1). There was no mucosal or skin pigmentation elsewhere on the body.

**Figure 2:**
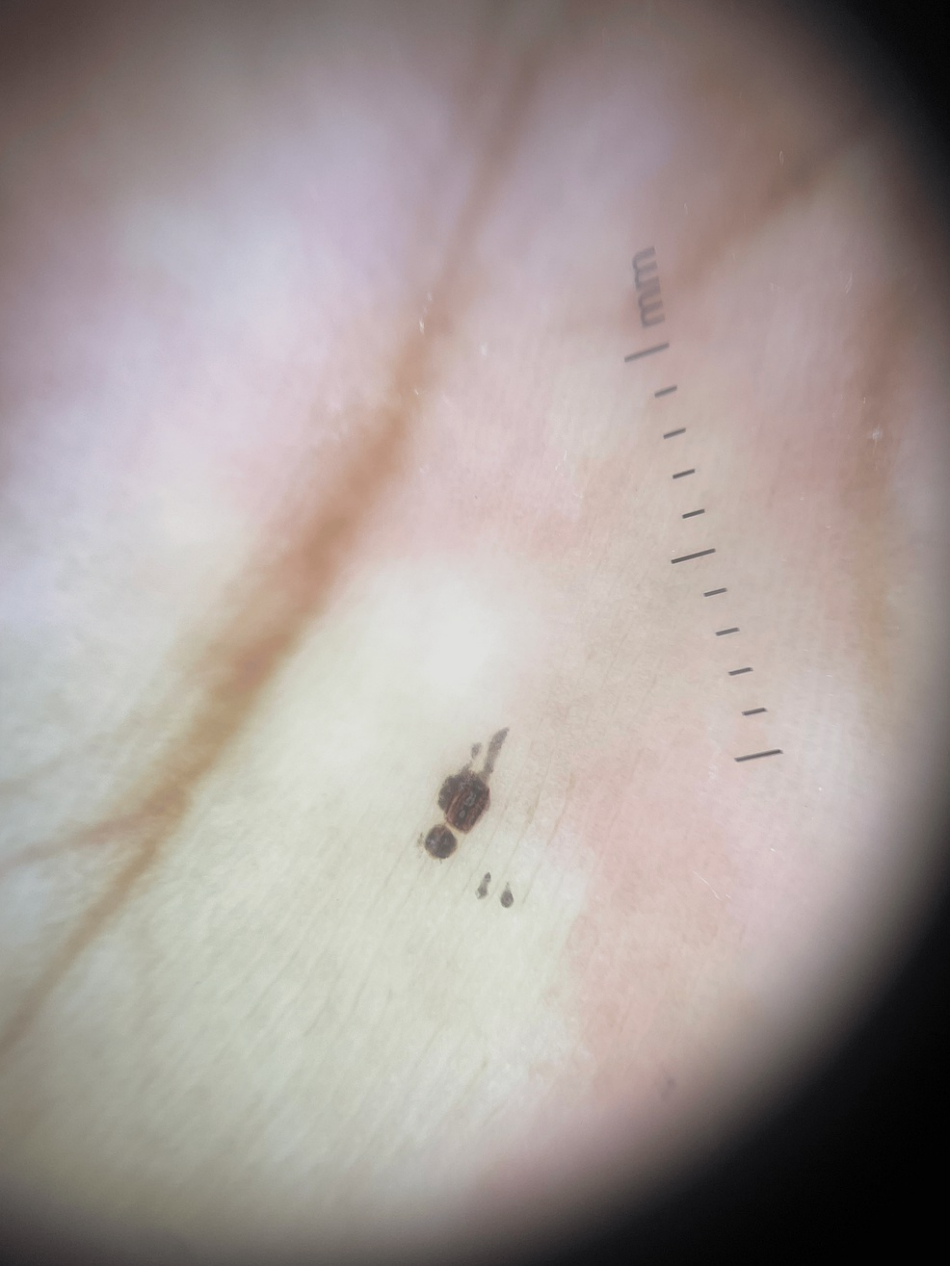
Dermoscopic image showing superficial black clods with streaky pattern spread along dermatoglyphics

On dermoscopy, superficial black clods and clusters with intact dermatoglyphics and a few clods with streaky patterns spread along the dermatoglyphic, giving a "stuck-on" appearance, were noted (Figure 2).

Lentigines, melanocytic nevi, tinea nigra, postinflammatory hyperpigmentation, dermatitis neglecta, and resolving petechiae were kept as clinical differentials. They were ruled out due to the absence of preceding lesions, the absence of associated local and systemic symptoms, and the superficial nature of pigmentation noted on dermoscopy. Dermoscopic differentials were junctional nevi, which show parallel furrows and lattice-like pigmentation; acral lentigines, which show moth-eaten appearance; and petechiae with reddish to violaceous dots.

Due to a history of sudden asymptomatic lesion onset during the rainy season and a change in accommodation, the differential diagnosis included cydnidae pigmentation. Notably, dermoscopy revealed features of superficial streaky pigmentation. However, conflicting with previous case reports, the absence of pigment reduction after degreasing with acetone posed a contradiction. Therefore, we decided to wait for spontaneous clearance of pigmentation, which eventually occurred after 10 days. This clearance served as another positive indicator for cydnidae pigmentation in our case.

## Discussion

Cydnidae pigmentation is the appearance of sudden, asymptomatic brown- or black-colored discoloration caused by contact with odorous hydrocarbonate secretions produced by special glands located in the thorax of cydnidae bug, *Chilocoris assmuthi* [2,3]. Pigmentation is due to the crushing of the insects and the accumulation of pigment in the skin furrows [4]. Cydnidae bugs mainly live in soil or sand but are also seen in vegetation-rich areas and near human dwellings, with a tendency to proliferate in the rainy season [5]. Clinical lesions mainly occur on exposed parts of the body. As time goes on, pigmentation darkens for the first few days and then slowly fades on its own over a period of one to two weeks without any residual effect. Most of the time, it can be rubbed off with acetone but not with soap and water [5].

This report shows dermoscopic findings of cydnidae in Indian skin. We are reporting this novel disorder of pigmentation with the asymptomatic sudden appearance of black-ink-like stains on palms, contradicting previous case reports for its reduction after degreasing with acetone and with its unique dermoscopic findings.

This may not be a rare clinical scenario but mostly goes underreported, so the sudden appearance of black-ink-like marks on the palms or face should alert us to the possibility of pigmentation caused by a substance secreted by cydnidae bugs.

The treatment only provides reassurance and hand-holding for the patient throughout the process of pigmentation fading on its own over a period of one to two weeks. Therefore, vigilant history-taking and a high index of suspicion are required.

## Conclusions

This may not be a rare clinical scenario but goes underreported, so the sudden appearance of black-ink-like marks on the palms or face should alert us to the possibility of pigmentation caused by a substance secreted by cydnidae bugs. The treatment only provides reassurance and hand-holding for the patient throughout the process of pigmentation fading on its own over a period of one to two weeks. Therefore, vigilant history taking and high index of suspicion is required. Superficial nature of pigmentation on dermoscopy aids in the diagnosis of this condition.
